# Application of Real-Time Tissue Elastography with a Low Frequency Convex Array Probe: A Noninvasive Approach to Differential Diagnosis of Liver Tumors

**DOI:** 10.1155/2014/378243

**Published:** 2014-04-01

**Authors:** Juan Wang, Hong Ai, Long Guo, Lifang Tan, Huilin Gong, Wei Wei, Litao Ruan

**Affiliations:** ^1^Department of Ultrasonography, The First Affiliated Hospital of Medical College, Xi'an Jiaotong University, Shaanxi 710061, China; ^2^Department of Pathology, The First Affiliated Hospital of Medical College, Xi'an Jiaotong University, Shaanxi 710061, China

## Abstract

To evaluate diagnostic performance of real-time tissue elastography (RTE) with a low frequency convex array probe for distinguishing benign from malignant hepatic tumors through trans-abdominal examination, elasticity images of 210 liver tumors were obtained by EUB-7500 (Hitachi Medical Systems and 3.5 MHz probe) and eventually 121 liver tumors were analyzed in the study. Elasticity images were classified into four types, from type a to d. Regarding type a or b as benign tumors and type c or d as malignant ones, sensitivity, specificity, and accuracy were calculated and the consistency between the findings of RTE and the pathohistological diagnosis was evaluated. The sensitivity, specificity, and accuracy were separately 97.2%, 88.0%, and 93.4% (*P* < 0.001). Moreover, there was a good consistency between the findings of RTE and the pathological diagnosis (kappa value 0.86). Among elasticity images of all the malignant tumors, the hepatocellular carcinomas (HCCs) mainly appeared in type c, and liver metastatic cancers in type d. Thus, RTE utilized as a novel noninvasive imaging examination method enables us to distinguish benign from malignant liver tumors. Moreover, it provides certain information for the differential diagnosis between HCCs and liver metastatic cancers.

## 1. Introduction

The accurate differential diagnosis for hepatic benign and malignant tumors, including that between hepatocellular carcinomas (HCCs) and liver metastatic cancers, is a significant factor for whether the patient ought to receive an operation. Among the noninvasive diagnosis methods, real-time tissue elastography (RTE) has been paid more attention because its specific principle differs from other methods. The image of RTE is analyzed according to the stiffness of lesion tissue, closer to the pathohistological diagnosis of the lesion, enabling us to acquire information more objective and accurate about the liver tumors. The tissue shape will change once compressed. The difference of change can respond to the differential strain of the tissue and then be colored in the imaging system. Two types of compression could be accessible in practical operation. The one is outer compression from the probe while the other is inner compression from rhythmic beating of heart [1, 2]. Recently RTE has already been applied to the clinical practice as a promising imaging method in the diagnosis of some superficial tumors such as breast cancer [3, 4], thyroid cancer [5, 6], and prostate cancer [7, 8]. However, its application was limited in deep tumors such as liver tumors due to the low tissue penetrating power of conventional linear array probes with high frequency. With the development of technology, convex array probes with low frequency have been integrated with RTE, making it possible to obtain clear strain images for the lesion located in the deep tissue. The aim of this study is to evaluate the value of RTE with a low frequency convex array probe (4–8 MHz) for differential diagnosis of hepatic benign and malignant tumors through transabdominal examination.

## 2. Materials and Methods

### 2.1. Study Design

**Figure 1:**
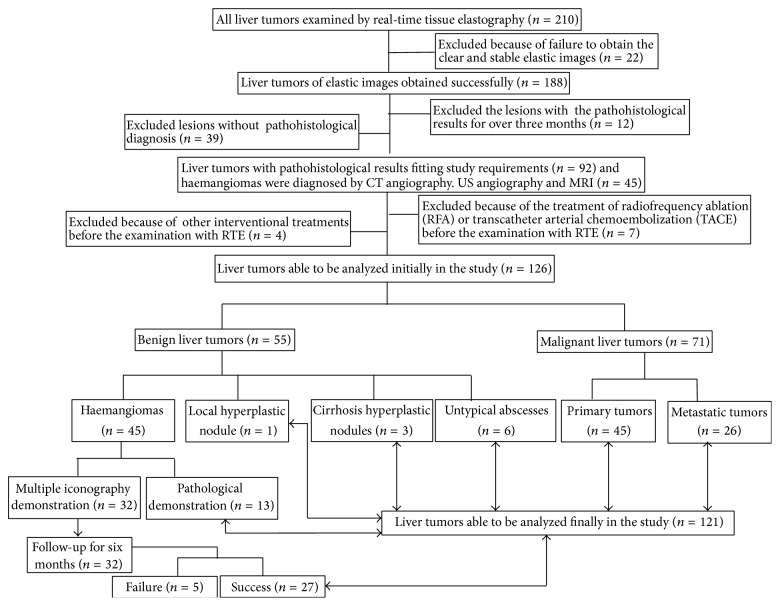
The flow diagram gives an overview of the study.

The patient in the study was recruited consecutively and the data was analyzed retrospectively. All patients with liver tumors, without ever receiving the treatment of radiofrequency ablation (RFA), transcatheter arterial chemoembolization (TACE), or other interventional treatments before RTE examination, can be accepted in this study. Regarding pathologic diagnosis as the reference standard, the gap time between the RTE and pathological examination for liver tumors is controlled in less than three months. The study was approved by the ethical guidelines of the Helsinki Declaration and our institutional review board. The flow diagram (Figure 1) gives overview about this study.

### 2.2. Patients

From October 2010 to October 2011, 210 liver tumors were consecutively performed by RTE with a low frequency convex array probe (4–8 MHz). All the examinations were performed on the agreement of the patients or the patients' family members. According to the criteria of study design, eventually, elasticity images of 121 liver tumors in 115 patients were analyzed (Figure 1). The mean age of the examined patients (72 men and 43 women) was 68 years (age range 22–81 years). The mean diameter of benign lesions was 43.2 mm and that of malignant lesions was 53.7 mm (the diameter of the lesion measured by B-mode ultrasonography). 3 patients with HCCs were complicated with liver cirrhosis nodules, and 3 patients with HCC were complicated with haemangiomas. 21 of 39 patients with HCC were positive for hepatitis B virus surface antigen, and 8 patients were positive for hepatitis C virus surface antigen; 4 patients were positive in both. As for 6 untypical liver abscesses without clinical symptoms of fever and chills, their two-dimensional ultrasonic images all showed heterogeneous and parenchymatous echolevel, irregular shape, and obscure boundary. According to the results of ultrasonography, of 121 liver tumors, 95 were located in the right lobe of the liver, 22 in the left, and 4 in the both right and left. 10 patients suffering haemangiomas represented abdominal mass and pain.

### 2.3. Pathologic Diagnoses

Pathological diagnoses of all lesions were performed by a pathological physician, Huilin Gong, who has worked in pathological department for 10 years, having rich experience at histopathological diagnosis for different types of liver tumors. The lesion samples were obtained from liver biopsy under the guidance of the US or liver resection. To avoid getting necrotic part of lesion in needle biopsy, the operator (Hong Ai, Director of Ultrasound Diagnosis Department, having 26 years' working experience) tried to obtain three tissue strips from different parenchymatous parts of each lesion. Some proportion of haemangiomas without pathological confirmation were diagnosed by patients' histories and are combining multiple iconographic examinations, including US, US angiography, 64-slice multidetector-row computed tomography (CT), CT arteriography, and magnetic resonance imaging (MRI). Then, we did follow up this part of haemangiomas for half a year.

### 2.4. Acquisition of Elastic Images

All the patients underwent RTE. Examination using this device did not require any additional instrument. The patients were examined in supine position with both arms elevated above the head. The process of obtaining RTE image is shown as follows. First, the liver tumors were clearly displayed under B-mode US and color Doppler US (EUB-7500, Hitachi Medical Systems and 3.5 MHz probe) to observe the tumors' position, size, shape, inner echolevel, boundary, and blood condition. Second, shifting into RTE model (EUB-7500, Hitachi Medical Systems and 4–8 MHz probe) and both the elasticity images and the B-mode images were displayed meanwhile. The range of region of interest (ROI) included the lesion tissue and its surrounding liver tissue as a contrast, avoiding nearby blood vessels, gallbladder, or bile ducts. While the size of the tumor was too large or existed part of necrotic tissue (fluid echo) within it, we chose one part of the parenchymatous instead of necrotic area of the tumor. Third, the patient was asked to hold the breath to prevent the ROI from interference with the movement of diaphragma, and then we chose the images by a series of operation such as freeze, playback, and so on. The RTE examination lasted approximately 5–10 min per patient separately. In the whole process, we did not need to exert any compressive force on the probe and only keep the probe contacting skin constantly, as the compression comes from the rhythmical beat of heart and great vessels, which is subtly different from the operation of examination for superficial tumors such as breast tumors [3]. All the echographic measurements were made by ultrasound diagnosis physicians, (Dr. Wang and Dr. Tan) who were blind to the pathological results of all lesions before RTE examinations. Ahead of this study, this technique of RTE has been also applying into the study relevant to the evaluation of liver fibrosis, and Dr. Wang was in charge of the RTE examination [9].

### 2.5. Analysis of Elastic Images

The movies recording elastic images of each lesion were replayed to select one stable frame to analyze. Normal liver tissue in this frame should colored by homogeneous green. To objectively analyze elasticity images of liver tumors in different types, we referred to the Tsukuba Elasticity Score which is a standardized scoring system for breast lesions by categorizing patterns of elasticity image of breast tumors into five classes from malignancy to benign [3]. Additionally, to minimize the interference from B-model images for imaging analyzer, each elasticity and its corresponding B-model image was separated by a computer cut system from one figure. Then, the categorizations of all elasticity images were performed by two doctors together who were blind to the final pathological diagnosis and routine ultrasound images for each lesion. (Both Dr. Wei and Dr. Guo).

**Figure 2:**
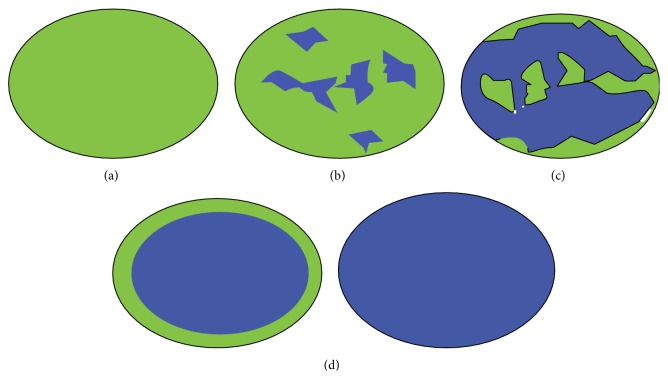
Ideograph presents general appearance of lesions for elasticity types of a, b, c, and d for liver tumors. Type d shows the whole lesion represents homogeneously blue with or without a green halo. Black circle indicates outline of lesion tissue on B-mode images.

According to the different distribution of colors shown in all lesions in this study, the categorizations of liver tumors are four types as follows: type a, the entire lesion has even strain, presenting homogeneous green; type b, the lesion has certain strain in most areas, meanwhile accompanied by some no strain areas (showing a mosaic pattern which is dominant by green); type c, the lesion has no strain in most areas meanwhile accompanied with certain strain in some areas (showing a mosaic pattern characterized by dominant blue area); type d, the lesion has no strain, showing the whole lesion represents homogeneously blue with or without a green halo. To identify it in more visualized effect, an elasticity ideograph of liver tumors in different types was designed (Figure 2).

### 2.6. Statistical Analysis

The detailed pathologic and elastic information of 121 lesions in 115 patients is shown in Table 1. Taking pathology of the lesion as golden standard, we considered type a or type b as benign tumor and type c or d as malignant tumor with the elasticity types of different liver tumors, and then calculated their sensitivity, specificity, and accuracy by the chi-square test (SPSS 16.0 version). *P* value less than 0.05 was considered to be statistically significant difference. Kappa value was applied into evaluating the consistency of the findings of RTE with the pathological diagnosis.

## 3. Results

### 3.1. Pathological Diagnosis

Table 1 illustrates the results about pathological diagnosis of 121 live tumors. All primary tumors including 39 HCCs and 6 intrahepatic cholangiocarcinomas (excluded severe 5 patients with hepatic cirrhosis representing diffuse nodules and 4 patients with ascites), 8 liver metastatic tumors, 3 Cirrhosis hyperplasic nodule, one local hyperplasic nodule, and 13 liver haemangiomas were diagnosed by pathohistology of tumor samples obtained from liver resection. All liver abscesses and 18 liver metastatic tumors (excluded 3 patients from the patients with diffuse liver cancers) were identified by liver biopsy. 27 hepatic haemangiomas were diagnosed by CT angiography and US angiography or MRI, which performed follow-up for half a year.

### 3.2. Elasticity Types

**Figure 3:**
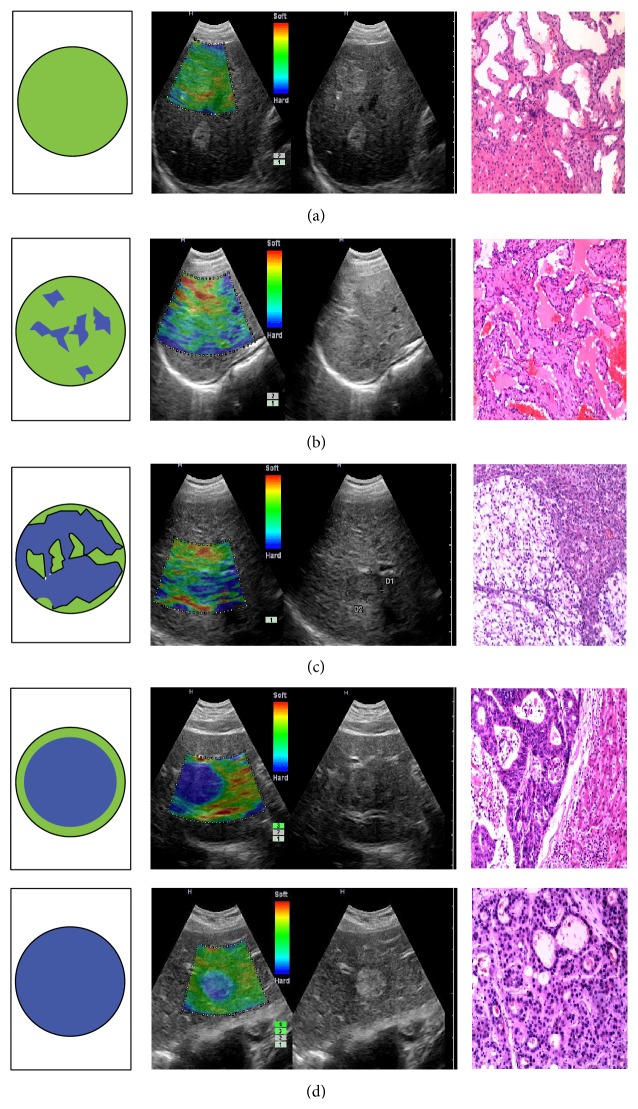
Images present elasticity ideographs, elasticity images, B-mode images, and corresponding pathological pictures, respectively. (a) 1: Haemangioma with type a elasticity in a 61-year-old man and the entire lesion is shown as homogeneously green. (b) 2: Haemangioma with type b elasticity in a 45-year-old woman and the lesion shows a mosaic pattern with dominant green areas. (c) 3: Hepatocellular carcinoma with type c elasticity in a 58-year-old woman and the lesion shows a mosaic pattern with dominant blue areas. (d) 4: Metastatic liver tumor from colorectal adenocarcinoma with type d elasticity in a 50-year-old man and the entire lesion shows homogeneously blue with a green halo surrounding. (d) 5: Hepatocellular carcinoma with type d elasticity in a 67-year-old woman, and the entire lesion shows homogeneously blue.

In each elasticity image of 121 lesions, the contrastively surrounding hepatic tissue was displayed as homogeneously green (some within small red) irrespective of their histological findings (there were 14 cases with chronic hepatitis and 19 cases with liver cirrhosis). Table 1 gives detailed information on four elasticity types from 121 liver tumors. In terms of 25 lesions in type d, 8 lesions from metastatic tumors presented homogeneously blue with whole green halos or parts of the green halo. Liver tumor images of four elasticity types were given in Figure 3.

### 3.3. Statistical Findings

Table 2 shows sensitivity, specificity, and accuracy of the diagnosis criteria with elasticity type for benign tumor and malignant tumor. The results were considered to be statistically significant difference (*P* < 0.001). The kappa value with measurement of agreement was separately 0.86. Additionally, Table 2 shows sensitivity, specificity, and accuracy of the diagnosis criteria with elasticity types for HCC and metastatic tumor, regarding type c as HCC diagnosed and type d as metastatic tumor.

## 4. Discussion

As a novel and noninvasive technique on the base of conventional B-mode scanner, RTE was applied in clinical setting to visualize space occupying lesions of liver in this study. Of 210 lesions, 188 (89.5%) could successfully obtain clear and stable elasticity images. In reality, Koizumi et al. [10, 11] has utilized successfully the same technique of RTE into evaluating liver fibrosis in patients with chronic hepatitis C. Furthermore, in their studies, liver stiffness of patients with ascites can be measured with RTE as well and even has the potential of being superior to transient elastography. However, in our study, 4 lesions from patients with ascites (the depths from peritoneum to liver surface are, resp., 2.4 cm, 3.1 cm, 2.8 cm, and 3.9 cm) did not obtain stable elastic image or failed to color-coded altogether. The detailed reason is unknown.

In terms of patients suffering diffuse liver cancers, it maybe brings inaccurate elasticity information of tumor tissue, since the area of surrounding normal liver tissue is not enough to be chosen as contrast, which means that the area of ROI includes most of tumor tissue and a small part of surrounding liver tissue, leading to the remarked increase of average stiffness analyzed. Consequently, the elastic information difference between the cancer tissue and its surrounding liver tissue was relatively minimized. Similarly, the elasticity images of patients with serious hepatic cirrhosis representing diffuse nodules also would be impacted. Of 12 unsuccessful images, there are 3 images from the patients with diffuse liver cancers and 4 images from diffuse cirrhosis hyperplasic nodules. Additionally, the depth of lesions would interfere with the stability of elasticity images obtained, showing different colors in different time. 5 lesions were located on the surface of the hepatic envelope and 4 lesions were too far away from the envelope of liver (the average depth was 6.7 cm, approaching to septum). Therefore, it is important for one operator to adjust the distance of the lesions from the heart through rotating or displacing probe. With considering a great possibility of severe necrotic tissue existing within the lesions, which might induce an inaccurate elastic information of lesions in reality, we tried to choose the parenchymatous (hyperechoic, hypoechoic, iso-echoic or hetero-echoic) instead of necrotic area (fluid echo) of the tumor as a contrast with its surrounding liver tissue. Of course, despite taking this method, it is still inevitable to be influenced by a small necrotic area of the parenchymatous tissue. Last but not least, it should be pointed out that a clear B-model image of each lesion is a prerequisite for its elasticity image successfully performed by RTE. The patients with severe fat infiltration failed to obtain stable elasticity images (two patients diagnosed by US in this study).

Kato et al. [12] applied the technology of RTE with a high frequency probe into differential diagnosis of intraoperative liver malignant tumors and developed the new score system named elasticity type of liver tumor (ETLT). Using this new criterion can help us distinguish rather accurately between HCC and metastatic adenocarcinoma. For different stiffness values of various liver tumors [13], the elasticity images in our study demonstrate four types (Figure 2) which concur with Kato's ETLT. RTE is also helpful for differential diagnosis of HCC and metastatic cancers (Table 2), and the characteristic of colorful distribution and strain in metastatic cancers concur with Kato's, mostly representing no strain in typed (the lesion was homogeneously blue) and small proportion (8/26) representing no strain in most areas with some areas of strain (the lesion had a mosaic pattern with blue area dominant). However, the feature of HCC in our study mainly presented type c, rather than type b previously reported by Koichi Kato. Compared with Kato's intraoperative performance, an important advantage in our study is without manumotive compression towards the probe. The technology of RTE with 4–8 MHz probe in our study is able to visualize images of liver tumors by transversely scanning abdominal wall. Moreover, another study by Inoue et al. [14] on freehand RTE of live lesion in an intraoperative setting also reported similar effect mimicking “visual palpation”. Besides clinical research, an animal experiment [15] applying combined hand-held B-mode/strain imaging on rabbit hepatic metastases also provided valuable evidences for detection of liver metastases that might be missed by standard B-mode imaging alone.

In our study, the results of Table 2 showed higher sensitivity (87.2%) and lower specificity (65.6%) of type c as HCC, nevertheless, lower sensitivity (69.2%) and higher specificity (86.7%) of type d as metastatic tumor. The reason may be that some kinds of metastatic tumors represented similar stiffness with HCCs, leading to reduction of specificity of Type c as HCCs. Additionally, HCC tended to represent lower stiffness than most of metastatic tumors, therefore type d as metastatic tumors showed higher specificity.

## 5. Limitation

**Figure 4:**
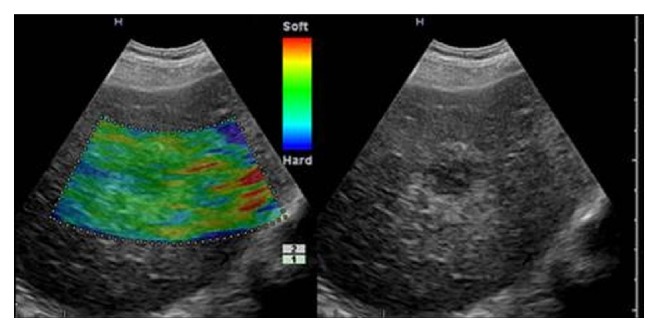
Haemangioma with type a in a 51-year-old woman and the entire shows homogeneously green.

**Figure 5:**
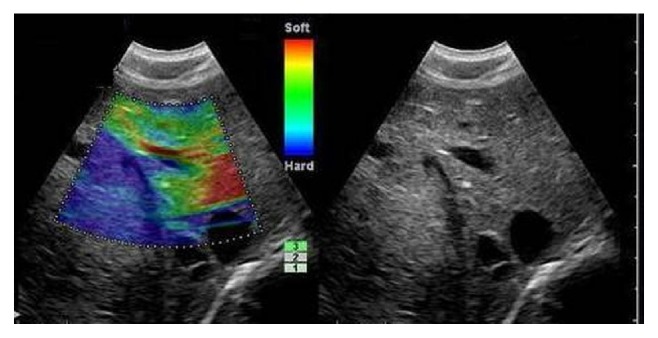
Local hyperplasic nodule with type d in a 15-year-old boy and the lesion shows homogeneously blue.

In this study, there was a limitation that patients with cancer were overrepresented because our hospital serves as a comprehensive one, where more patients with cancer tend to visit. Therefore, the findings cannot necessarily be extended to the general population. Furthermore, amongst benign lesions, haemangiomas occupied predominantly (40/50), which have softer tissue compared with other types of tumors, such as Local hyperplasic nodules and cirrhosis hyperplasic nodules (Figure 4 represents type a and Figure 5 represents type d). Therefore, further study about detailed elastic information of liver cancers from different resources assessed by RTE need to be continued.

Overall, RTE utilized an ultrasound probe with a low frequency making it possible to clearly visualize the liver tissue through the thoracoabdominal wall, which provides a valuable information for the differential diagnosis of liver tumors. More importantly, the elasticity image obtained by RTE can lead to a prediction of pathological diagnosis of liver tumors, helping us objectively and accurately recognize information of tumor tissue, and this technique is considered as a new and noninvasive approach differentiating from other conservational ultrasonography methods. Besides, without any help of assistant advice, elasticity image obtained by RTE on the base of traditional B-mode ultrasound can show B-mode image at the same time, which is of a good assistance in detecting the region of lesions that are difficult to be recognized by the B-mode scanner alone. Furthermore, it offers a certain help for the diagnosis of those untypical masses under the US as well, such as untypical abscess, haemangioma. Figure 4 was one case in this point.

In conclusion, with the development of the technique of RTE, the range of its utilization has become larger. As a new tool for differential diagnosis between benign and malignant liver tumors, although it is evaluated by visual, it provides certain diagnosis value for different types liver tumors. Surely, there would be more objective information, if a quantity way of RTE, strain ratio, was utilized into diagnosis of liver tumors. RTE will be a promising method as used in the diagnosis of liver tumors.

## Figures and Tables

**Table 1:** Elasticity type and pathological diagnosis of live tumor.

Pathological diagnosis	Elasticity type of live tumor				
	*n *	Type a	Type b	Type c	Type d
Benign tumor					
Haemangioma∗	40	23	15	2	0
Local hyperplasic nodule	1				1
Cirrhosis hyperplasic nodule	3	1	1	1	0
Untypical abscess	6	0	4	2	0
Malignant lesion					
Primary tumor					
HCC	39	0	2	34	3
ICC	6	0	0	3	3
Metastatic tumor					
Colorectal adenocarcinoma	7	0	0	0	7
Pancreatic adenocarcinoma	5	0	0	1	4
Gastric adenocarcinoma	6	0	0	2	4
Lung cancer	3	0	0	2	1
Gallbladder cancer	5	0	0	3	2

Total	121	24	22	50	25

HCC: hepatocellular carcinoma; ICC: intrahepatic cholangiocarcinoma. ∗27 of 40 Haemangiomas were diagnosed by CT angiography, US angiography, and MRI.

**Table 2:** Sensitivity, specificity, and accuracy of the diagnosis criteria with elasticity type for benign tumor and malignant tumor, hepatocellular carcinoma, and metastatic tumor.

Diagnosis Criteria	Sensitivity (%)	Specificity (%)	Accuracy (%)
Type a or b as benign tumor; type c or d as malignant tumor	97.2 (69/71)	88.0 (44/50)	93.4 (113/121)
Type c as HCC	87.2 (34/39)	65.6 (21/32)	77.5 (55/71)
Type d as metastatic tumor	69.2 (18/26)	86.7 (39/45)	80.3 (57/71)
